# EZ-SEP: Extended Z-SEP Routing Protocol with Hierarchical Clustering Approach for Wireless Heterogeneous Sensor Network

**DOI:** 10.3390/s21041021

**Published:** 2021-02-03

**Authors:** Zhanserik Nurlan, Tamara Zhukabayeva, Mohamed Othman

**Affiliations:** 1 Faculty of Information Technology, L.N. Gumilyov Eurasian National University, Nur-Sultan CO 010000, Kazakhstan; zhaskazak@mail.ru; 2 Department of Communication Technology and Network, Universiti Putra Malaysia, Serdang 43400, Selangor, Malaysia; 3 Laboratory of Computational Science and Mathematical Physics, Institute of Mathematical Research (INSPEM), Universiti Putra Malaysia, Serdang 43400, Selangor, Malaysia

**Keywords:** Z-SEP, SEP, LEACH protocol, WSN, WMN, WMSN

## Abstract

Wireless sensor networks (WSN) are networks of thousands of nodes installed in a defined physical environment to sense and monitor its state condition. The viability of such a network is directly dependent and limited by the power of batteries supplying the nodes of these networks, which represents a disadvantage of such a network. To improve and extend the life of WSNs, scientists around the world regularly develop various routing protocols that minimize and optimize the energy consumption of sensor network nodes. This article, introduces a new heterogeneous-aware routing protocol well known as Extended Z-SEP Routing Protocol with Hierarchical Clustering Approach for Wireless Heterogeneous Sensor Network or EZ-SEP, where the connection of nodes to a base station (BS) is done via a hybrid method, i.e., a certain amount of nodes communicate with the base station directly, while the remaining ones form a cluster to transfer data. Parameters of the field are unknown, and the field is partitioned into zones depending on the node energy. We reviewed the Z-SEP protocol concerning the election of the cluster head (CH) and its communication with BS and presented a novel extended mechanism for the selection of the CH based on remaining residual energy. In addition, EZ-SEP is weighted up using various estimation schemes such as base station repositioning, altering the field density, and variable nodes energy for comparison with the previous parent algorithm. EZ-SEP was executed and compared to routing protocols such as Z-SEP, SEP, and LEACH. The proposed algorithm performed using the MATLAB R2016b simulator. Simulation results show that our proposed extended version performs better than Z-SEP in the stability period due to an increase in the number of active nodes by 48%, in efficiency of network by the high packet delivery coefficient by 16% and optimizes the average power consumption compared to by 34.

## 1. Introduction

Wireless sensor networks have garnered increased attention worldwide and become one of the most emerging technologies because of the revolutionary advancements in IoT, IoE and VANET in terms of size, deployment cost and user-friendly interfaces together with machine-to-machine communication (M2M) to support this eco-system, for development of which WSNs usage affects a lot [1,2,3,4].

In recent years, it has become easy to develop cost-effective sensors using wireless sensor network technology. These devices are very small—even tiny—in size, inexpensive, with straightforward processing and computational capabilities and affordable compared to previous generation sensors. They are capable and very useful for sensing the entire environment, collecting information from fields, processing and transmitting aggregated data to the user with reliable quality of service [5]. Moreover, they have sufficient ability to define the physical environment in detail and control each other when they are selected as observers and combined with other nodes [6].

For efficient data acquisition, large-scale WSNs often need to be partitioned to improve the scalability, energy efficiency and load balancing [7]. Therefore, the unfolding of nodes is divided into two types: (1) a uniformly distributed field and (2) a randomly distributed field. In a uniformly distributed field, the nodes are systematically arranged in a predefined way [8], while in a randomly distributed field, the sensor nodes are randomly deployed in the area. In any case, due to the large number of nodes, network management issues such as connection management and fault detection become complex.

In WSNs, the amount of nodes can exceed a hundred or even a couple of thousand, therefore, this number is too large for the base station to control them for the most efficient routing, or to place them in a specific location to program their location at each node. In addition, this kind of nodes are usually so small and cheap that they are not always equipped with GPS or optimized antennas. This means that collaboration is an important key to solving and ensuring “low power consumption” when delivering data to the base station. Indeed, these nodes will have to be able to communicate with each other, provided that there is no established network infrastructure and a predetermined location of the nodes [9].

The sensor node environment in the area of the wireless sensor network may be realized in two different ways: (1) uncontrolled environment and (2) controlled environment [10]. In an uncontrolled environment, sensor node location is not pre-defined, thus, the accessibility of a sensor node is hard to establish, so these nodes are not regularly monitored. In a controlled environment, on the other hand, every sensor node position is easily accessible and frequently monitored.

The topology and infrastructure of a typical WSN consists of a base station and nodes located in a zone. Nodes sense events in an environment, process and aggregate data and then transmit it to a base station in direct or in multi-path mode, delivering records from one node to another in the network [11]. Nodes require high energy to deliver the sensed data to a base station if a message is sent directly, so their resources can be exhausted quickly. To solve this problem and extend the lifespan, data sensed is transferred from one node to the next and to the base station last, in multiple paths, so the energy consumption is reduced. Methods with multiple-sink have also been proposed to balance power consumption and reduce data transmission latency [12].

Energy efficiency is the most challenging task in wireless sensor networks. Therefore, the development of an energy-efficient routing algorithm makes the research work in this area very interesting. This and many more and more protocols such as LEACH [13] and its derived protocols, SEP [14], I-SEP [15], SEP-V [16] as well as all derivatives and enhanced forms of the SEP protocol, ESRA [17], HEED [18], and DEEC [19] are developed to increase network lifetime. Hierarchy methods have been also used to save the energy in sensor nodes. This method clusters the nodes, where data aggregation is performed on the cluster head (CH). Hierarchical routing protocols provide significant energy savings for WSNs. In a hierarchy-based routing algorithm, clusters are formed, and each cluster is assigned a head node (CH) using a certain threshold value *T(n)*.

The clustering technique is also effective in minimizing network traffic to the base station and prolonging the lifespan of the network [20]. Cluster networks are of two types: (1) a homogeneous sensor network, in which each node is assigned an identical initial energy value, and (2) a heterogeneous sensor network, where the nodes differ in initial energy from each other [21,22]. In a homogeneous network, cluster heads remain static after their selection [13]. Those cluster heads can remain operational for the entire life of the network. As the cluster heads perform data aggregation and then transmit that aggregated data to the remote long-distanced base station, they are constantly overloaded. Thus, they expire first and result the instability in the network [23]. In a heterogeneous network, instability in the network performance occurs after the first node death [24]. To fix these issues and improve the network efficiency, lifetime and stability period, various routing algorithms have been proposed.

### 1.1. Parent Protocol Analysis

Z-SEP is a zonal stable election protocol for a wireless heterogeneous sensor network, in which nodes differ in initial power. In this algorithm, the connection of nodes with the sink was carried out in a hybrid way: (1) direct communication and (2) communication through the cluster head. Z-SEP offers two types of nodes: (1) regular normal nodes that are located near the base station, and (2) extended advanced nodes that are located at a distance from the BS. The initial energy of normal nodes is lower than that of advanced nodes. The deployment field area is limited in scope and divided into three zones: (1) zone 1, (2) zone 2 and (3) zone 3. Normal sensor nodes are placed in zone 1. Some of the advanced nodes are located in zone 2 and some in zone 3. Normal nodes are connected to base station directly and communicate, send data in direct mode, whereas the extended advanced nodes form the cluster heads and the cluster heads aggregate records from their member nodes. The cluster heads collect the aggregated data and transmit it to the BS. In the unrestricted area, as the field length changes, the communication of the extended advanced nodes with the BS becomes more difficult. In this case, nodes that are distant from base station, will consume energy a lot. Consequently, the network lifetime will be reduced. This will also affect the selection of the cluster head among the extended advanced nodes. In short, the Z-SEP protocol is discussed briefly below:

- Direct communication with the base station requires a lot of energy. So far, the sensor node battery capacity and computing capabilities are limited. Clustering and cluster head elections are generated only on extended advanced nodes. CHs aggregate and collect sensed records from their member nodes and transfer it to BS. Consequently, network instability arises from high energy consumption as these nodes are quickly depleted. Consequently, the entire service life of the WSN is reduced.
- It is a heterogeneous routing protocol in which the heads of the cluster are randomly elected from among the advanced nodes, wherefore, the probability that an advanced node with low residual energy, which is quickly depleted and leads to network instability, will be elected as the head of the cluster is quite high. Instability arises when the first head node of the cluster dies. This leads to imbalance in the cluster. Moreover, there is a possibility that there will be no cluster head left for that cluster when a huge amount of nodes die out.
- The instability of the sensor network in the Z-SEP protocol can also be caused by the fact that with an increase in the size of the deployed area of the field, the head nodes of the cluster will start to die out faster, since they would consume more power to deliver data to the sink. Since the distance and energy consumption are directly proportional to each other. In addition, because regular normal nodes and cluster heads in extended advanced nodes communicate directly with the base station.

### 1.2. Contribution Preview

In this paper, we considered that the BS has no energy limits; the size of the unfolded area is unknown; low initial energy nodes are located close to the BS, and high powered sensor nodes are located distant. With this model in mind, we have proposed novel algorithm well recognized as the EZ-SEP protocol. In EZ-SEP, normal nodes will communicate with the base station directly, while cluster members in advanced nodes will pass the sensed data to the cluster head, and aggregated records will be sent to BS by the cluster head. Furthermore, cluster head election at advanced nodes will depend on a threshold value based on the node’s residual energy. This model affects the reduction of the cluster head power consumption and the increase in the sensor network service life.

In the first round, each node, as in LEACH, selects a random number in the range from 0 to 1. If the selected number is less than the threshold, then this sensor node is elected as the cluster head for this contemporary round. In the end of the 1^st^ round, the remaining residual energy of each node is revised. If a node has higher remaining residual energy, then this sensor node will have a better chance of becoming a cluster head for the next round. This method increases the lifetime of the first node to its death, which is an important factor in the stability of the network. Moreover, the behavior of EZ-SEP will be measured with the previous parent algorithm taking into consideration such evaluative cases as (1) change in the position of the base station (2) change in density of nodes in the field and (3) change in the initial energy of the nodes. Simulation results show that the proposed EZ-SEP algorithm improves the network stability and also extends the lifespan over existing cluster-based heterogeneous parent protocols.

### 1.3. Paper Structure

Paper structure proceeds as following: Section 2 contains a literature review and the necessary background information. Our proposed framework and system module are described in Section 3. Simulation results and analysis are discusses in Section 4. Section 5 presents a comparison of EZ-SEP with parental protocols. Finally, the whole research work and paper are concluded in Section 4.

## 2. Related Work

The main purpose of sensor nodes in the WSN is to sense the area of the environment, collect data, process and aggregate it and deliver it to the base station. Direct transmission is the simplest way for nodes to communicate with a base station or sink. However, they need a lot of power to forward records. Thus, their resources will be quickly depleted and the node will quickly die out due to unnecessary energy use [25]. One way to reduce power consumption is to send data from one node to another and finally to the BS. The most important issue in wireless sensor networks is an energy efficiency. Thus, routing protocols with an energy efficiency solution represent an engaging research area in this area. The main goal of these algorithms is to reduce the power consumption of nodes in the network.

Gandomi et al. in [15] proposed the I-SEP (IoT-SEP) protocol, introducing a threshold energy value *T(n)* for each node type for the SEP protocol, where depending on this energy threshold, the existing cluster head and its corresponding cluster members must either be changed or stay transmitting in the next round. Thus, an effective method for electing the head of the cluster is being studied. The nodes in this network are of three types: (1) regular normal nodes, (2) intermediate nodes, with energy value between normal and advanced nodes, and (3) advanced nodes. At each round, remaining residual energy *E_res_* of the cluster head is weighed up. If *E_res_* is lower than the threshold value *T(n)*, then the new cluster head selection procedure is initiated and clusters are renewed. Thus, additional energy consumption when forming a new cluster is reduced.

The cluster head selection procedure is the same as in LEACH and SEP algorithms. By considering the probabilities of each node, the threshold value *T(n)* for cluster head election is specified for each node type. Thus, *p_(N)_, p_(I)_* and *p_(A)_* are considered as probabilities for regular normal, intermediate and extended advanced nodes, respectively as:

(1) $$p_{\left(N\right)}=\frac{p}{1+a\alpha +b\beta }$$

(2) $$T(n_{N})=\left\{\begin{array}{c} \frac{p_{\left(N\right)}}{1-p_{\left(N\right)}\left(r\;mod\frac{1}{p_{\left(N\right)}}\right)},\;\;if\;n_{N}\in G_{1}\\ 0,\;\;otherwise \end{array}\right.$$

(3) $$p_{\left(I\right)}=\frac{p\left(1+\beta \right)}{1+a\alpha +b\beta }$$

(4) $$T(n_{I})=\left\{\begin{array}{c} \frac{p_{\left(I\right)}}{1-p_{\left(I\right)}\left(r\;mod\frac{1}{p_{\left(I\right)}}\right)},\;\;if\;n_{I}\in G_{2}\\ 0,\;\;otherwise \end{array}\right.$$

(5) $$p_{\left(A\right)}=\frac{p\left(1+\alpha \right)}{1+a\alpha +b\beta }$$

(6) $$T(n_{A})=\left\{\begin{array}{c} \frac{p_{\left(A\right)}}{1-p_{\left(A\right)}\left(r\;mod\frac{1}{p_{\left(A\right)}}\right)},\;\;if\;n_{A}\in G_{3}\\ 0,\;\;otherwise \end{array}\right.$$

where *a* and *b* are the set of advanced and intermediate nodes. *G*_1_, *G*_2_ and *G*_3_ denotes the set of nodes in each type of normal, intermediate and advanced nodes respectively, that had not assigned as a cluster head in former epochs, and *r* is the current round. Nodes have *E*_0_, *E*_0_(1 + *β*) and *E*_0_(1 *+ α*) as the initial energies of normal, intermediate and advanced nodes respectively.

Once the cluster head is assigned, the remaining member nodes in the cluster join the cluster head according to the information sent in the advertisement (ADV) message. In this way, each round cluster heads, clusters and its members regularly change. Once node is elected as a cluster head, according to traditional SEP, this node cannot take a part in a cluster head selection procedure next 1*/p* epochs.

The next research paper focusing on energy-based threshold sensitive cluster head election protocol is a method called improved threshold-sensitive stable election protocol (ITSEP) as described by Zhao. This protocol enhances the threshold formula by taking into consideration the node distance from the base station, the number of its neighbor nodes, nodes remaining residual energy and the nodes-to-nodes average distance [26].

Therefore, the algorithm introduces the average distance between nodes and nodes residual energy into a threshold, so that a node with higher energy and closer to the base station will become a cluster head. Thus, the improved threshold formula is expressed as:

(7) $$T\left(s\right)=\left\{\begin{array}{c} \frac{p}{1-p\times \left(r\;mod\left(\frac{1}{p}\right)\right)}\times \omega_{i},\;\;if\;s\in G\\ 0,\;\;if\;s\notin G \end{array}\right.$$

where *r* is the round number, *i* is node ID, *ω_i_* is

(8) $$\omega_{i}=C_{1}\times \frac{1}{d_{\left(i\right)}}+C_{2}\times \left(1-\frac{1}{Number_{\left(i\right)}}\right)+C_{3}\times \left(\frac{S\left(i\right).E}{E_{ave}\times K_{opt}}\right)+C_{4}\times \left(\frac{1}{d_{C_{i}-BS}}\right)$$

where *S(i).E* is the nodes residual energy, *d_Ci-BS_* is the node distance from the base station, *Number_(i)_* is the number of nodes in *d*_0_ range, *d_(i)_* is an average distance between node *i* and neighbor nodes, *E_ave_* is the node average energy, *K_opt_* is a constant value. *C*_1_ is the control parameter of the relative distance between nodes, *C*_2_ is the degree of the node, *C*_3_ is the nodes residual energy and *C*_4_ is the node distance to the base station [26].

Another algorithm, focusing on the distances from sensors to the base station, that optimally balances the energy consumption among the sensors, is described in [27]. It’s a distance-based threshold for distributed cluster head election, where the authors propose LEACH with distance-based thresholds, called LEACH-DT, in which the probability of a node to become a cluster head depends on its distance to the base station or the sink:

(9) $$T_{i,r}=\left\{\begin{array}{c} \frac{p_{\left(i\right)}}{1-p_{\left(i\right)}\times \left(r\;mod\frac{1}{p_{\left(i\right)}}\right)},\;\;if\;G_{i}\left(r\right)=0\\ 0,\;\;if\;G_{i}\left(r\right)=1 \end{array}\right.$$

At the beginning of each group of rounds, *G_i_(r)* is set to 0 for [1*/p*] successive groups, to ensure that a node becomes a cluster head once in every group of rounds of [1*/p_i_*] rounds. Base station initially evaluates the distance based on the nodes signal strength to estimate the probability *p* and broadcasts it to all the nodes. The nodes then autonomously make decisions without any centralized control.

The authors in [28] also introduce an improved threshold assignment algorithm using node distance from BS. They determine smaller *p* for distant nodes from base station so that distant clusters will be bigger in size and distant nodes will have less opportunity to be selected as a cluster head than closer nodes to BS. Before first setup, base station broadcasts a packet declaring a “start” of network task. Sensor nodes receive this packet including signal strength and estimate the distance from BS. Nodes then send their distances to BS by a carrier sense multiple access (CSMA) protocol. Therefore, base station evaluates the maximum and minimum distances in the network. Thence, base station broadcasts these two packets to all the nodes in the wireless network.

Thus, new probability *p* for cluster head election:

(10) $$p_{new}\left(n\right)=p\;\left(1-\alpha \left(\frac{d_{n}-d_{M}}{d_{max}-d_{M}}\right)\right)$$

is for when *p_new_(n) <* 1, and 1 for otherwise. Where, *p* is the probability to choose a node as a cluster head, *d_n_* is the distance of *n*-th node from BS and *d_m_* is an average distance:

(11) $$d_{m}=\frac{d_{min}+d_{max}}{2}$$

and α is:

(12) $$\alpha =\frac{d_{max}-d_{min}}{d_{max}}$$

and a new threshold assignment with consumed energy value used in threshold is:

(13) $$T\left(n\right)=\frac{p}{1-p\;\left(r\;mod\left(\frac{1}{p}\right)\right)}\times \left[\frac{E_{n,\;current}}{E_{n,\;max}}+\left(r_{n,\;s}\mathrm{div}\left(\frac{1}{p}\right)\right)\left(1-\frac{E_{n,\;current}}{E_{n,\;max}}\right)\right]$$

where *E_n,current_, E_n,max_* are residual and initial energy of *n*-th node, *r_n,s_* is consecutive rounds in which *n* has not been a cluster head and *r* is a number of rounds.

When we to talk about clustering algorithm, then Heinzelman et al. in [13] presented a protocol known as low energy adaptive cluster hierarchy (LEACH). It is a cluster-based routing protocol for homogenous networks. In LEACH, the cluster heads rotate randomly to evenly distribute the energy load between the sensor nodes. In setup phase clusters are formed and CH are selected, while in steady-state phase sensed data is aggregated and delivered to the sink. Cluster head is selected as following: nodes randomly select a number *r* between 0 and 1. If *r* is less than the threshold value *T(n)*, then this node becomes a cluster head for the current round. Threshold is calculated as:

(14) $$T\left(n\right)=\frac{p}{1-p\times \left(r\;mod\left(\frac{1}{p}\right)\right)},if\;n\;\epsilon \;G\;$$

where *G* is a set of nodes that are associated in cluster head election and which are not picked out as a cluster head in the past 1*/p* rounds. Once node is elected as a cluster head then it cannot be elected as a cluster head in the next successful 1*/p* rounds. Therefore, the possibility of other nodes to be elected as a cluster head is increased. The elected cluster head broadcasts an ADV message to remaining nodes, so that non-cluster head nodes choose and form a cluster based on the received message signal strength. After receiving a feedback message from nodes, cluster head creates a time division multiple access (TDMA) time schedule, then sets a time slot to every member node of its cluster, representing a time they are allowed to send a data. In this manner at each round setup phase is replaced by steady-state phase and continues until the lifetime of the network [29].

However, the LEACH algorithm does not give a good performance in heterogeneous environment. The stable election protocol (SEP) was presented as a cluster-based two level heterogeneous-aware routing algorithm consisting of normal regular and extended advanced nodes [14]. In a SEP algorithm, the network becomes unsteady after the death of the first node. Therefore, to improve the stability period, SEP provides even-tempered power consumption technique. Thus, sensor nodes with higher initial energy have better possibility to become a cluster head than normal nodes due to weighed election probability based on remaining residual energy. Hence, network lifetime and energy consumption is well-balanced [30]. Normal nodes have *E_0_* initial energy, while advanced nodes have *E*_0_(1 + *α*). Scene *α* indicates more energy than a normal node. Thus, the total network energy is *n(*1 *− m)E*_0_ *+ nmE*_0_*(*1 *+ α) = nE*_0_*(*1 *+ αm)*. Hence, the network is enhanced by 1 *+ αm* energy. To refine the period of stability, the new calculated epoch is 1*/p_opt_ (*1 *+ αm)*, since the network has *αm* of more sensor nodes and accordingly *αm* times more power. Each node selects a random number between 0 and 1 and if this number is less than the threshold value *T(s)*, then it becomes a cluster head for the current round. This cluster head cannot be nominated for the same epoch at this round. Threshold value *T(s)* increases as rounds rise and becomes equal to 1 at the round of last. It means that, the probability of remaining nodes to be elected as a cluster head is 1 at the last round. So the probability *p_nrm_* of normal nodes and *p_adv_* of advanced nodes to become a cluster head is:

(15) $$p_{nrm}=\frac{p_{opt}}{1+\alpha m}$$

(16) $$p_{adv}=\frac{p_{opt}}{1+\alpha m}\left(1+\alpha \right)$$

where *p_opt_* is a cluster head probability and an average *n · p_opt_* cluster heads must be elected per round per epoch. Advanced nodes have a higher chance to be selected as a cluster head than normal nodes. *m* is the subset of extended advanced nodes, where *α* is the energy factor that is added to the whole network. Thus, the threshold value for normal and advanced nodes is:

(17) $$T(n_{nrm})=\left\{\begin{array}{c} \frac{p_{nrm}}{1-p_{nrm}\times \left(r\;mod\frac{1}{p_{nrm}}\right)},\;\;if\;n_{nrm}\in G\prime \\ 0,\;\;\;otherwise \end{array}\right.$$

(18) $$T(n_{adv})=\left\{\begin{array}{c} \frac{p_{adv}}{1-p_{adv}\times \left(r\;mod\frac{1}{p_{adv}}\right)},\;\;if\;n_{adv}\in G\prime \\ 0,\;\;\;otherwise \end{array}\right.$$

where *G’* is non-cluster head nodes. SEP algorithm performs better than the LEACH, since advanced nodes extra energy is consumed in balance, thus enhancing the network stability period.

Node heterogeneity is another interesting challenge for researchers. The authors in [31] presented a routing algorithm with hybrid link communication for heterogeneous WSN, named zonal-stable election protocol (Z-SEP), where the concept of zonal field deployment of nodes in the network is introduced. In addition, nodes communication is implemented in hybrid mode, where normal nodes communicate with the sink or base station directly, while advanced nodes are clustered and transmit data to BS through cluster heads. Simply saying, nodes in the deployed network area are separated into three fields: normal nodes are allocated in zone 1 and advanced nodes are placed in zone 2 and zone 3. Sensor nodes in zone 1 deliver data to the base station directly, whereas nodes in zone 2 and zone 3 via cluster head. As in SEP *m* is a subset of entire nodes *n* with *α* times extra energy. Thus, (1 – *m)n* of normal nodes. *K_opt_* is an optimum number of cluster heads. And according to SEP algorithm, cluster head probability is *p_opt_ = K_opt_*:

(19) $$p_{opt}=\frac{K_{opt}}{n}$$

The threshold value for cluster head selection in Z-SEP is as in LEACH protocol:

(20) $$T\left(n\right)=\left\{\begin{array}{c} \frac{p_{opt}}{1-p_{opt}\left(r\;mod\frac{1}{p_{opt}}\right)},\;\;if\;n\in G\\ 0,\;\;\;if\;n\notin G \end{array}\right.$$

where *G* indicates sensor nodes which are not assigned as a cluster head in the past 1*/p_opt_* rounds. Thus, the probability for advanced nodes to be elected as a cluster head is:

(21) $$p_{adv}=\frac{p_{opt}}{1+\alpha m}\left(1+\alpha \right)$$

and the threshold value is:

(22) $$T\left(adv\right)=\left\{\begin{array}{c} \frac{p_{adv}}{1-p_{adv}\left(r\;mod\frac{1}{p_{adv}}\right)},\;\;if\;adv\in G\prime \\ 0,\;\;\;otherwise \end{array}\right.$$

where *G’* denotes nodes that are not assigned as a cluster head in the last 1*/p_adv_* rounds. Z-SEP has the same cluster formation procedure as in parent protocols like SEP and LEACH, where the cluster head election, further its message broadcasting and cluster members respond to cluster head are based on RSSI signal and the data transmission to cluster head is based on TDMA schedule. Clustering is not performed in regular normal nodes because they have lower initial value in energy than in extended advanced nodes, since the energy consumption in clustering is high enough. Normal nodes die quickly if clustering is performed among them, resulting a bad stability period. Therefore, they are assembled near to BS and communicate with it directly, while advanced nodes are far from BS because of their higher energy. So, vise a versa, advanced nodes will exhaust faster if data transmission to BS is done in direct mode, because energy consumption is high due to long distance. Thus, to save energy in advanced nodes, clustering method is applied.

However, Z-SEP does not take into account the remaining residual energy of nodes when choosing it as a cluster head, thus there is a possibility that a node with a low residual energy will be chosen as a cluster head in the upcoming round, which ultimately can lead to a rapid depletion of the selected cluster head and an imbalance of the entire network.

## 3. Proposed Work

### 3.1. Communication Model

**Figure 1:**
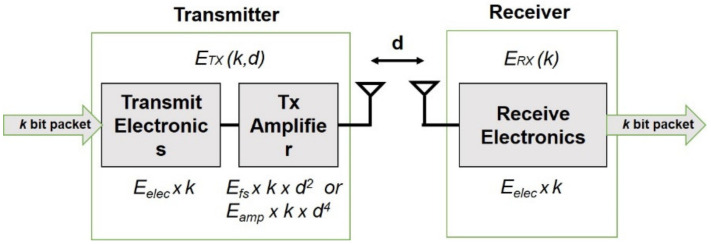
Radio communication model.

The communication module given in Figure 1 is considered as a study case in proposed protocol.

If the distance between the cluster head and its associate node is short, then the free-space model is considered, otherwise if the distance is longer, then the multipath fading model is used [15].

The power consumed to transmit *k* bits packet of data to the sensor node allocated *d* distant away can be given as:

(23) $$E_{TX}\left(k,d\right)=E_{TX\_elec}\left(k\right)+E_{TX\_amp}\left(k,d\right)\;E_{TX}\left(k,d\right)=\left\{\begin{array}{c} E_{elec}*k+E_{fs}*k*d^{2},\;\;\;d\leq d_{0}\\ E_{elec}*k+E_{amp}*k*d^{4},\;\;\;d>d_{0} \end{array}\right.\;E_{RX}\left(k\right)=E_{RX\_elec}\left(k\right)+kE_{elec}\;d_{0}=\sqrt{\frac{E_{fs}}{E_{amp}}}$$

where *d* is the Euclidian distance from the sending node to the receiving node, *E_elec_* is the receiver/transmitter energy consumption per bit, to receive *k* bits packet of data E_elec_*k amount of energy is spent on the radio module, *E_fs_* and *E_amp_* are the free space and the multi-path fading amplifier energies respectively.

### 3.2. Proposed Threshold

The proposed protocol is a hierarchical clustering heterogeneous routing protocol known as extended Z-SEP routing protocol with hierarchical clustering approach for wireless heterogeneous sensor network (EZ-SEP). This algorithm is an extended version of Z-SEP algorithm, that prolongs the lifetime of the sensor network. We improve the threshold value *T(n)* of the parent protocol to residual energy-aware cluster head selection algorithm. Thus, the new modified *T(n)* equation is derived as:

(24) $$T\left(n\right)=\left\{\begin{array}{c} \frac{p}{1-p\times \left(r\;mod\left(\frac{1}{p}\right)\right)}\times \frac{E_{res}}{E_{0}}\times K_{opt},\;\;for\;n\in G\\ 0,\;\;\;for\;n\;\notin G \end{array}\right.$$

where *E_res_* is the nodes remaining residual energy, and *E_0_* is the initial level of the supplied energy. The optimal cluster number *K_opt_* can be given as in [32]:

(25) $$K_{opt}=\sqrt{\frac{n}{2\pi }}\sqrt{\frac{E_{fs}}{E_{amp}\times d^{4}\left(2m-1\right)\times E_{Tx,\;Rx}-mE_{DA}}}\times M$$

where *n* is the node number, *d* is the distance to the base station, *m* is the quantity of cluster heads associates, *E_Tx,Rx_* is the tranceiving/receiving energy, *E*_DA_ is the data aggregation energy, *E*_0_ is the assigned initial power of each sensor node and *M* denotes the diameter of the deployed network.

Nodes in the network spend definite amount of energy after the data transmission, which varies according to the distance *d* between the transmitting and receiving node. The proposed protocol network structure and link communication are described as below:

### 3.3. Network Structure

**Figure 2:**
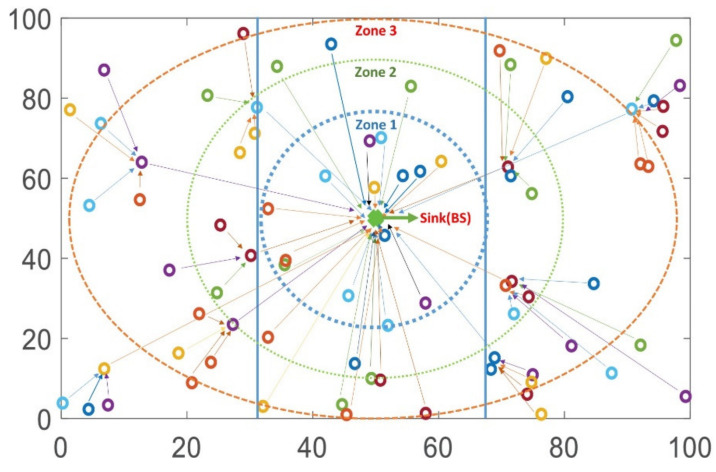
Nodes connection to base station.

Protocols usually use a randomly organized allocation of nodes in the deployed area, thus the inefficient use of nodes energy can be observed. In EZ-SEP algorithm, the deployed area is separated into three fields as zone 1, zone 2 and zone 3 to optimize the energy consumption at the nodes. To distribute the network load and due to nodes assigned initial energy value, nodes are categorized into two groups as normal and advanced nodes. Advanced nodes have more energy than normal nodes. Therefore, normal nodes are deployed in zone 1, close to base station, while advanced nodes are deployed distant to BS in zone 2 and zone 3. We suppose that nodes are not mobile in the field and field dimensional is unknown. Assume *m* as a fraction of total nodes *n* with *α* times more energy. Thus, (1 *− m)n* of normal nodes. Normal regular nodes transmit records to base station directly, whereas in advanced nodes hierarchical clustering is formed among nodes, where one sensor node is elected as a cluster head and other cluster members join and deliver data to this cluster head, and then this cluster head transmits aggregated data to BS as shown in Figure 2.

### 3.4. Link Communication

Link communication with BS is built in two ways in EZ-SEP protocol:

#### 3.4.1. Direct Communication

Zone 1 is equipped with normal nodes, since they have low energy and allocated close to BS. These nodes sense environment, then send collected data to BS in direct mode.

In the first round, clusters and cluster heads are formed using normal LEACH algorithm, where cluster head is elected using Equation (17) as in a SEP algorithm for normal nodes. After transmitting data, each sensor node in the network consumed a certain amount of energy, which is different for each node. The power consumption depends on the distance between the sending and receiving nodes, represented as *d*. Therefore, in the next round, cluster head is selected using the modified equation in Equation (24).

The cluster head sends its cluster head announcement information to member nodes in the corresponding cluster, once it is elected for the current round. Depending on this received message signal strength, other remaining non-cluster head sensor nodes decide whether to join this cluster head or not. Further, to avoid the data collision in the network, cluster head broadcasts and allocates TDMA schedules in the member nodes to transmit data in different timeslots. This process continues until the end of all rounds and until all the nodes in the network are exhausted and lost its energy. This time period is called Set-Up Stage.

#### 3.4.2. Cluster Head Communication

Zone 2 and zone 3 are equipped with advanced nodes, which have higher energy than normal nodes. These nodes use clustering technique to deliver records to base station. The advanced nodes form a cluster, and the cluster head is elected among those nodes in the cluster, while the remaining nodes become an element of this cluster. These cluster associates sense the environment and then transmit data to cluster head. Cluster heads collect the records, processes and deliver them to the sink.

Transmission of data to cluster heads occurs during the time slot allocation in each node. To save energy, only transmitting nodes remain active, while all other nodes in the cluster turn-off their radio. Cluster head will start the data processing, after the all nodes data transmission in the cluster is ended up. Cluster head receives the data and then aggregates it to remove any redundancy. In addition, compresses the data as much as possible for fair bandwidth utilization. Cluster head then forwards the data to the sink or BS. This stage is called Steady-State Stage.

**Figure 3:**
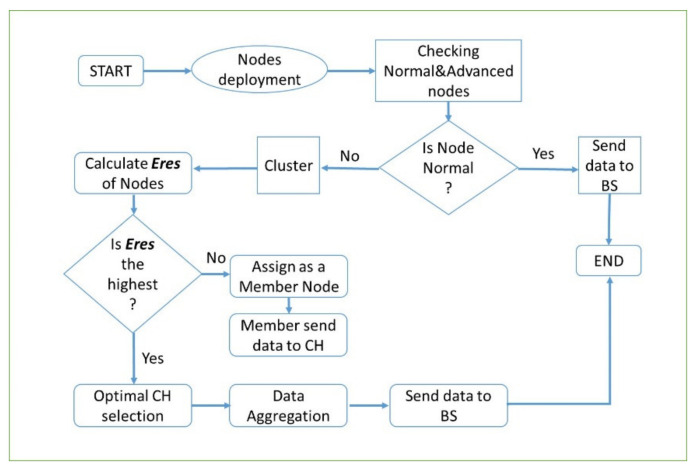
Communication processes of EZ-SEP protocol.

The entire above two processes are illustrated in flowchart as shown in Figure 3.

## 4. Simulation and Analysis

We model the proposed algorithm and parent protocols in this section. In addition, taken into consideration different evaluation scenarios and analyzed the output results of our protocol comparing with other given protocols in terms of performance behavior. Network model parameters considered for MATLAB simulations are described in Table 1. The packet size is supposed to be 4000 bits. One hundred nodes are placed in zone 1, zone 2 and zone 3, with base station allocated in the center of the field area *x* and *y*, as shown in Figure 2. *X* is an x-axis, whereas *y* acts for y-axis. *R_max_* indicates the quantity of total rounds in the specified network and is dependent on the application. Possible parameters are shown in Table 2.

*E_0_* is the assigned initial energy of each node, *E_amp_* is nodes amplification and receiving energy and *E_fs_* is the energy dissipated to transmit the data between two CHs and *E_DA_* is the consumed energy used throughout the data transmission latency. Data packet transmission and receiving energy is *E_TX_/E_RX_*. Using these parameters, EZ-SEP algorithm starts the network routing procedure improving the network parameters below:

(1) Increase in alive active nodes value
(2) Increase in package delivery rate
(3) Reducing the value of power consumption (residual energy)

**Figure 4:**
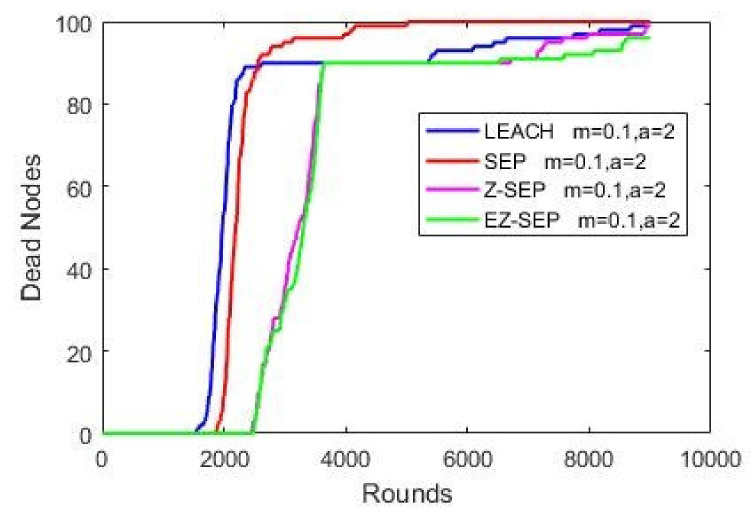
Dead nodes representation.

The above parameters are plotted against the quantity of active alive nodes, the package delivery rate and the remaining residual energy compared to the three WSN protocols. A red color line represents the SEP protocol, a blue color one the LEACH protocol, magenta color represents the parent Z-SEP algorithm and the proposed EZ-SEP algorithm is represented by a green color. The graphs in the figure illustrate that at the beginning of the network, the LEACH protocol demonstrates the worst performance in comparison with the other three. First node death and network instability in LEACH protocol begins at round 1508, while in SEP happened at 1816th round. The proposed EZ-SEP protocol at the beginning shows an approximately 5% improvement as the number of rounds reaches 2000 and a gradually increase over a parent Z-SEP protocol, but shows a better lifetime for the first node death at the 2497th round, while in Z-SEP the first node died at the 2461th round. The performance of proposed EZ-SEP protocol is improved after the 6000th round, showing a better and frequent stability period rather than the Z-SEP protocol. Generally speaking, the number of dead nodes in the EZ-SEP algorithm is less than in the other three protocols as the network continues to operate. Death of all nodes in parent protocols is considered at 8000 rounds, while the proposed EZ-SEP protocol shows improved results over 9000 rounds, thus significantly improving the whole network lifetime as shown in Figure 4.

**Figure 5:**
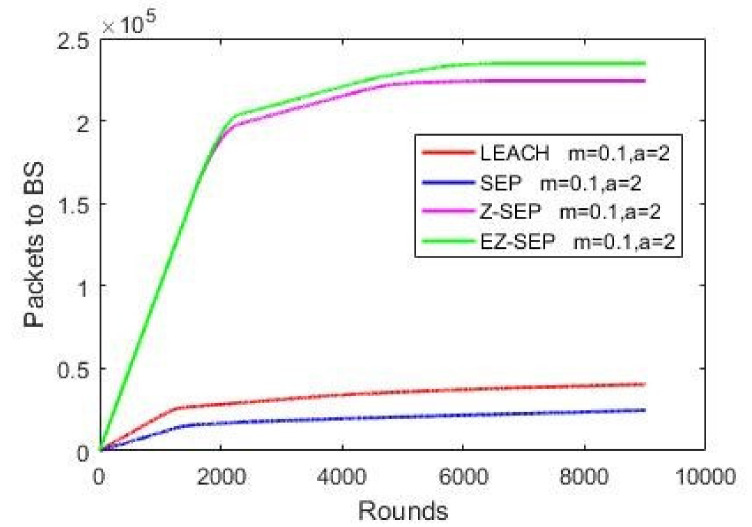
Packets delivery ratio to BS.

Network packet delivery ratio to a BS is illustrated in Figure 5. Nodes’ data transmission to a base station is the main role of a WSN. More data will be sensed and more environmental information is formed and delivered to the sink, if the network is alive for a long time, thus making the network efficiency more convenient for long time usage. Given below is the graph of all four protocols data packet delivery to BS. It is easily seen in the graph that proposed the EZ-SEP algorithm’s performance is much better than that of the other protocols by transmitting more than 2.4 × 10^5^ packets to BS. The other three compared protocols show their incapability to improve upon the proposed new algorithm, as illustrated in Figure 5.

**Figure 6:**
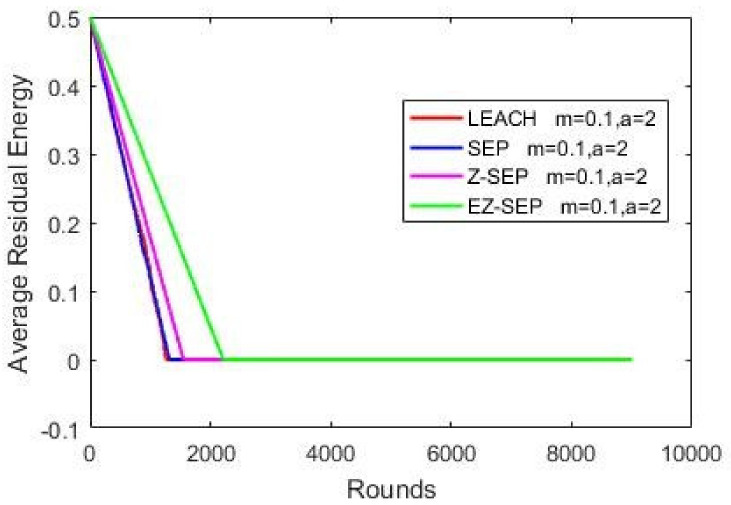
Nodes energy consumption.

Nodes’ energy consumption during network operation in terms of residual energy is illustrated in the graph below. In the graph, we can see that in terms of energy consumption and nodes residual energy, our proposed protocol displays better results compared with the other protocols. For a given number of rounds, the EZ-SEP algorithm provides lower power consumption. At the 1000th round, the nodes’ remaining residual energy is 0.3 joules for EZ-SEP, 0.2 J for Z-SEP and around 0.1 J for the LEACH and SEP protocols. The lowest energy consumption was shown by our proposed EZ-SEP protocol, which ultimately will be reflected in an increase in the number of live nodes and an increase in the number of delivered packets to the BS, as well as in increase of the network service lifetime and, accordingly, the efficiency of the network, as shown in Figure 6.

For further analysis and comparison of proposed EZ-SEP algorithm with the abovementioned three protocols, three different network conditions have been observed for parameters like dead nodes, package delivery rate as well as nodes’ power consumption in terms of residual energy. The supposed network conditions are as below:

### 4.1. Altering the Base Station Position

In this scenario, the base station position is changed and the network performance in our proposed protocol is observed in comparison with the other protocols. BS positions *sink.x* and *sink.y* are changed from 50 to 25, without modifying other parameters. These changes affect dead node performance, packet delivery rate and average power consumption. Simulation outputs show that these parameters display better results in the proposed EZ-SEP compared to the parent protocols, as demonstrated in Table 3(a). When the data routing is started, Z-SEP shows better performance in node life as compared to the proposed EZ-SEP, but the EZ-SEP algorithm improves after fa ew rounds and shows better results after 3000 rounds and this remains true till the end. The SEP and LEACH protocols show the worst results in comparison with the Z-SEP protocol and EZ-SEP. In addition, EZ-SEP is better in packet delivery ratio and transmits more packets compared to the three existing protocols. Furthermore, the LEACH and SEP algorithms demonstrate higher energy consumption compared to the other protocols. Nodes’ residual energy is around 0.1 J in the proposed EZ-SEP at round 2000, while in Z-SEP it is almost 0. LEACH is the most energy-consuming protocol of all. Thus, our proposed protocol increases the service lifetime and improve the stability period of the sensor network, since it consumes less energy than other ones.

### 4.2. Changing Field Density of Nodes

Node deployment in the field is changed in this scenario. Thus, a large quantity of sensor nodes are deployed in one portion of the area, in zone 1 for example, while the remaining few are placed in another part, in zone 2. The base station is located in the center with no change. Nodes can be skewed in the x-axis as well as in y-axis of the area. The proposed EZ-SEP protocol performance is evaluated under these conditions, compared with the remaining protocols. Results shown in Table 3(b) indicate that proposed EZ-SEP is in better condition than other protocols, if the nodes position in the network is altered. The whole sensor network behavior is changed. SEP and LEACH does not display good results in comparison with the Z-SEP and EZ-SEP protocols in terms of stability period of alive nodes. Z-SEP and EZ-SEP operate the same till the 2000th round, after which the proposed EZ-SEP has longer nodes’ lifetime and nodes’ stability than Z-SEP. In the data transmission ratio, SEP and LEACH deliver a significantly lesser amount of data than the proposed algorithm, while Z-SEP stays near to EZ-SEP. In any case, the EZ-SEP protocol gives better results than the parent protocol. In addition, our proposed protocol has a better energy consumption line in the graph, while LEACH is the most energy-consuming protocol than others.

**Table 3:** Different network condition comparison.

	Dead Nodes Representation	Packets Delivery Ratio	Nodes Energy Consumption
**Altering the Base Station Position** **(a)**	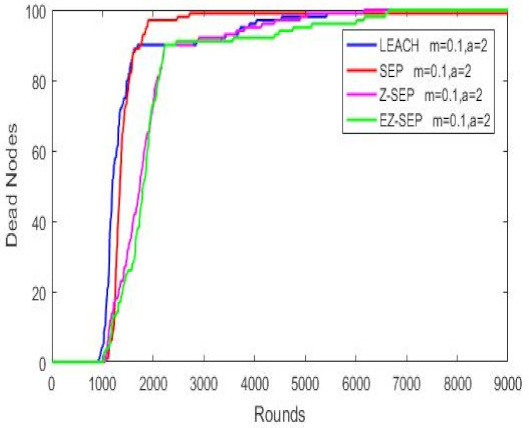	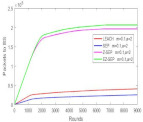	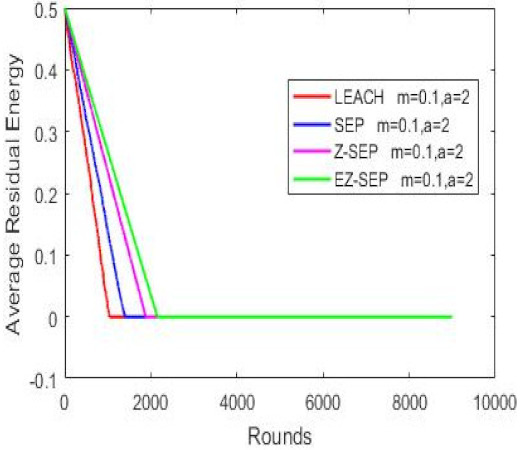
**Changing Field Density of Nodes** **(b)**	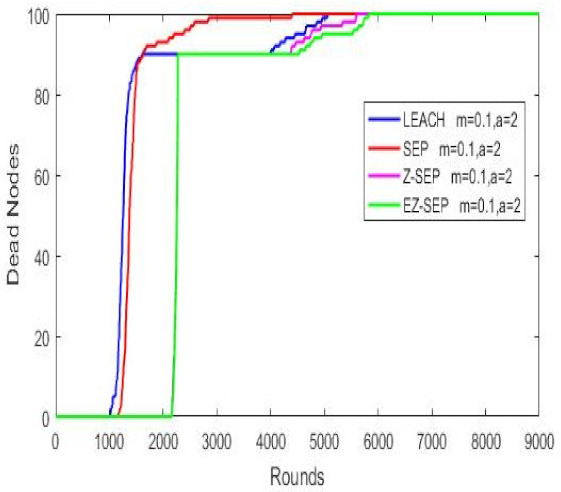	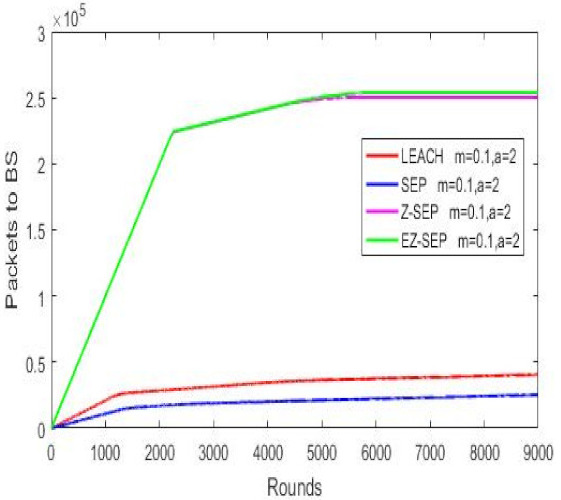	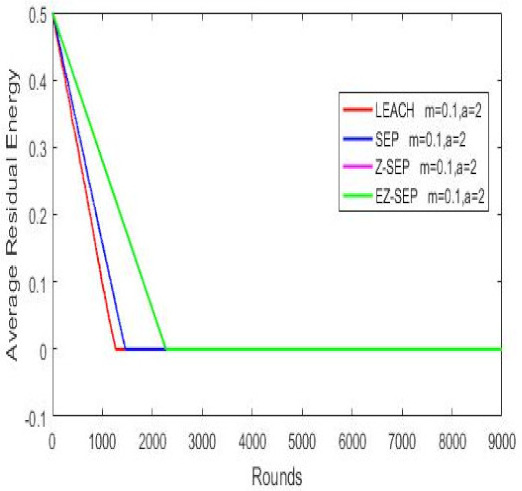
**Incrementing Nodes Initial Energy** **(c)**	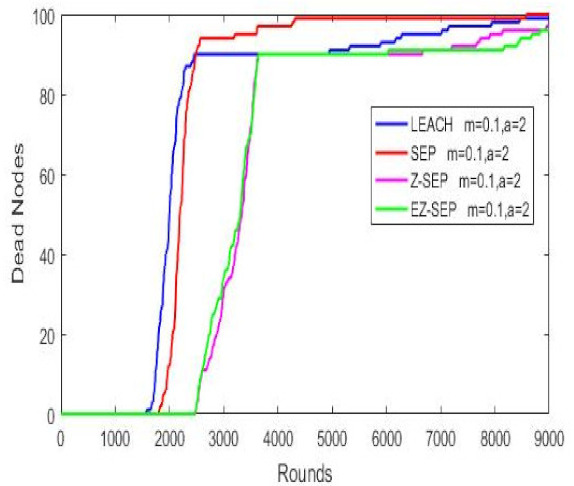	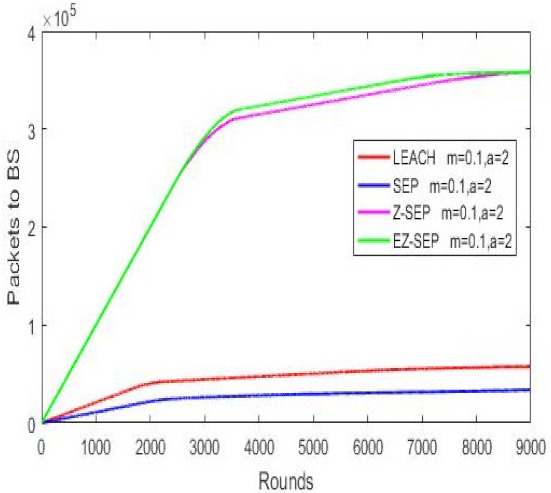	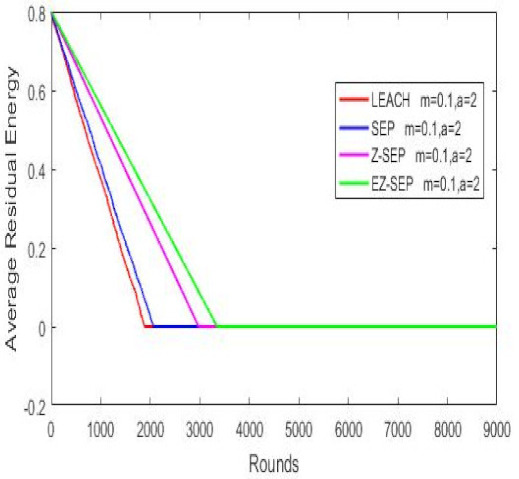

### 4.3. Incrementing Nodes Initial Energy

Nodes’ initial energy is the key factor in a WSN, since nodes are battery limited and this has the main role in whole network stability and performance and there is no possibility to recharge the battery once a node is placed in the field. We analyze the network behavior by increasing the nodes’ initial energy *E*_0_ from 0.5 J to 0.8 J and observe our proposed protocol compared to other algorithms. It is obvious that increased network energy will affect the lifetime and stability period. We analyze the influence of added energy in advanced nodes on the whole network system in the process of cluster head election and the data delithe very to BS, since advanced nodes consume more energy than normal nodes and advanced nodes have different energy from each other after cluster heads are elected. Cluster heads are then formed based on the remaining residual energy. In Table 3(c), the networks energy consumption is demonstrated. According to the graphs in the figure, it is seen that proposed EZ-SEP protocol gives better results compared to the other three protocols, when the nodes’ initial energy is changed. The lowest energy consumption is observed in EZ-SEP, while the highest is seen in LEACH. Nevertheless, as compared to the previous initial value, the new added value in SEP and LEACH gives an improved network lifetime. Thus, nodes are almost about to exhaust after the 2000th round, while previously they became exhausted twice as earlier at round 1000. The same is true for Z-SEP at round 3000, while in EZ-SEP it happens after 3500 rounds. In the case of nodes life, in the EZ-SEP protocol nodes show the longest stability period almost until the end of round 9000 and still remain alive after with first node death at round 2467, while SEP performs the worst as illustrated by all the nodes being dead at the 4000th round and the death of the first node at round 1707. In LEACH, the first death occurs at round 1610, which is the worst index. The parent protocol Z-SEP has its first node death at the 2489th round, which is the best index, but is much more unstable than the proposed EZ-SEP. Thus, the proposed algorithm is the best when nodes have added initial energy. In data transmission ratio, EZ-SEP shows more packets delivered in the 2500–8500 rounds range and is close with Z-SEP afterwards, which means that EZ-SEP sends much more data in total than the Z-SEP protocol.

### 4.4. Comparative Analysis of the Observed Protocols

In this section, we determine the performance parameters of the proposed EZ-SEP protocol, which is the extended version of Z-SEP algorithm. Various properties and evaluations mentioned before for the above studied protocols have been combined and listed in Table 4. Network level means energy homogeneity or heterogeneity. Energy efficiency is the energy consumption index of the algorithm. Stability period is the time interval between the life and death of the first node. Scalability defines the scalability of the network.

SEP and LEACH show similar results to each other and are the worst compared to Z-SEP and EZ-SEP, on the other hand EZ-SEP performs better in all the parameters against them. According to the above parameters, EZ-SEP demonstrates better indexes than its parent protocols.

## 5. Conclusions

Energy efficiency and network lifetime are two major attributes in the design of a network routing algorithm. Constructing an energy-efficient protocol that distributes the network load is a challenge. Z-SEP is a useful algorithm for this, however it still has some weak points. The extended zonal stable election protocol (EZ-SEP) is a two level heterogeneous, hierarchical cluster-based routing protocol that provides a modified cluster head selection technique as well as nodes communication with the sink. Thus, the nodes in EZ-SEP transfer data to their BS in a hybrid manner, meaning normal regular nodes deliver records to the base station directly, whereas extended advanced nodes make use of a clustering mechanism. This is aimed at extending the lifetime of the network by taking into account the network energy dissipation. An improved routing process can be achieved by selecting the cluster head among the nodes based on the remaining residual energy in comparison to the parent Z-SEP protocol. This method increases the period of stability of the network before the death of the first node, as well as improving the number of live nodes in the network. Thus, the packet transmission ratio is enhanced, meaning that a bigger amount of environmental events are monitored, sensed and delivered to the base station. Simulation results show the improvement of network performance in parameters like lifetime, packets sent to BS and energy consumption.

In conclusion, three different protocols like SEP, LEACH and Z-SEP have been observed and compared to the proposed EZ-SEP algorithm, where EZ-SEP demonstrates better performance reducing the dead nodes by 48%, increasing the packet delivery ratio by 16% and decreasing the average power consumption by 34%.

## 6. Future Research

The proposed work can be strengthened by taking into consideration additional parameters in cluster head election procedure, and tested on other realistic scenarios. According to their physical structure, wireless sensor networks are similar to wireless mesh networks (WMNs), thus many researchers combine these two networks to obtain a more reliable network [33,34,35]. The routing procedure in these protocols acts in the same manner in these networks. In WSNs, data is aggregated from sensors sensing the environment [36], while in WMNs data is driven by the new connected client [37]. This is the main difference. Nodes in WMNs communicate to each other as in WSNs. They aggregate data from clients when they are in movement and change position, where this data is used to find the location of the client by triangulation or positioning method [38], as well as to define the number of connected clients in the network, which is used to evaluate the client density in the given network. Thus, WMNs can be extended to collect data from sensors for environmental monitoring applications [39]. Therefore, in the future, WSN routing algorithms integrated with WMNs forming wireless mesh sensor networks (WMSNs) can be studied and developed well and experimented with in realistic scenarios.

## Figures and Tables

**Table 1:** Simulation parameters.

Parameters	Value
Network diameter (M)	100 × 100 m^2^
Nodes (n)	100 nodes
*E_0_*	0.5 Joules
Receiving (*E_amp_*)	0.0013 pJ/bit/m^4^
Free space model (*E_fs_*)	10 pJ/bit/m^2^
Power amplifier (*E_amp_*)	100 pJ/bit/m^2^
Data aggregation (*E_DA_*)	5 nJ/bit
Tranceiving/receiving (*E_TX_/E_RX_*)	50 × 10^−9^

**Table 2:** Different parameters of the network.

Parameters	Value
Field axis *X*, *Y*	300 × 300 m^2^
Sink axis *X*	150 m
Sink axis *Y*	150 m
*R_max_*	9000
Cluster head associates (m), quantity	4

**Table 4:** Comparative table of various properties.

Protocols	Parameters			
	Network Level (Homo/Hetero)	Network Stability	Energy Efficiency	Scalability
SEP	heterogeneous	medium	low	medium
LEACH	homogenous	medium	very low	very low
Z-SEP	heterogeneous	high	medium	medium
EZ-SEP	heterogeneous	high	high	medium

## Data Availability

The data presented in this study are available on request from the corresponding author.
